# A high level of lncFGD5-AS1 inhibits epithelial-to-Mesenchymal transition by regulating the miR-196a-5p/SMAD6/BMP axis in gastric Cancer

**DOI:** 10.1186/s12885-021-08192-x

**Published:** 2021-04-23

**Authors:** Lin Liu, Cheng Zhang, Jizhao Wang, Xu Liu, Hangying Qu, Guangjian Zhang, Ting Liang, Jiansheng Wang, Jia Zhang

**Affiliations:** 1 The Department of Thoracic Surgery, The First Affiliated Hospital of Xi’an Jiaotong University, Xi’an, Shaanxi China; 2 The Department of Radiology, The First Affiliated Hospital of Xi’an Jiaotong University, Xi’an, Shaanxi China

**Keywords:** lncFGD5-AS1_1_, miR-196a-5p_2_, ceRNA_3_, Epithelial-mesenchymal transition_4_, Prognose of gastric Cancer

## Abstract

**Background:**

Long non-coding RNA (lncRNA) was a vital factor in the progression and initiation of human cancers. This study found a new lncRNA, FGD5-AS1, which can inhibit EMT process, proliferation, and metastasis in vitro and in vivo.

**Methods:**

qRT-PCR was employed to test the expression of lncFGD5-AS1 in 30 gastric cancer patients’ cancer tissue and para-cancer tissue. Overexpressed lncFGD5-AS1 cells shown sharply decrease of proliferation, migration, and epithelial-mesenchymal transition (EMT). miR-196a-5p/SMAD6 was confirmed as downstream molecular mechanism of lncFGD5-AS1 by expression correlation analysis and mechanism experiments. In vivo study illustrated overexpression of lncFGD5-AS1 suppression tumor growth.

**Results:**

LncFGD5-AS1 served as a ceRNA of miR-196a-5p to release its inhibition on SMAD6, a conventional inhibitor on the BMP pathway. Comparing with normal gastric cancer cells, FGD5-AS1 overexpressed group had fewer migration cells, lower cell viability, and lower EMT transformation rate. Meanwhile, xenografts nude mice injecting with overexpressed-FGD5-AS1 cells also shown smaller tumor weight and volume.

**Conclusion:**

In conclusion, this research supported the first evidence that FGD5-AS1 suppressed proliferation and metastasis in gastric cancer by regulating miR-196a-5p/SMAD6/BMP axis and suggested a potential therapeutic candidate for gastric cancer.

**Supplementary Information:**

The online version contains supplementary material available at 10.1186/s12885-021-08192-x.

## Background

According to the clinical research carried out in 2013, gastric cancer is the third most common and the third lethal tumor [1]. In various clinical research focused on mortality of gastric cancer, the metastasis markers pTNM, rN and positive lymph node ratio closely correlate with poor prognosis and shorter survival. Among those who die of gastric cancer, metastatic gastric cancer accounts for majority [2–4]. The significantly high lethality rate of metastatic gastric cancer can be due to the concealment of metastasis. Until now, clinicians still lack appropriate methods to mitigate post-metastatic gastric cancer because current operative treatment only gets considerable success in the early stage of gastric cancer [5–7]. Based on these reasons, a proper treatment point of metastasis gastric cancer is urgently needed.

On the complex development of tumor initiation to metastasis, cancer cells need to adapt to permanently changing and often hostile environmental conditions. To survive in these conditions, tumor cells lose their epithelial feature and change to mesenchymal traits. This transition mainly expresses as losing cell-cell tight junctions, apical basal cell polarity and spindle-like cell shape [8–10]. Meanwhile with these changes, tumor cells show high migration, invasion, and survival features. These features are the core of tumor associated epithelial–mesenchymal transition (EMT). Therefore, EMT has became a highlight point in cancer treatment research.

EMT is influenced by several molecular networks. Various extracellular stimuli can activate or inhibit EMT process and most of them are classical growth factors which most secreted by tumor cells. Besides that, signaling pathways, such as TGF/BMP, Wnt, JAK-STAT, AP-1, Notch, NF-κB and Hippo signaling also induce or modulate the EMT process [11–13]. As a pivotal element in EMT process, bone morphogenetic proteins (BMPs) belonging to the transforming growth factor -β (TGF-β) family binds to their receptors (BMPRs) followed by activating the phosphorylation and expression of small mothers against decapentaplegics 1/5/8 complex (SMAD1/5/8) in cell. Additionally, SMAD6, a protein that also belongs to small mothers against the decapentaplegic family, acts as a phosphorylated inhibitor playing a regulator role in BMP pathway [14, 15].Besides of SMAD family dependent working mode, several non-conventional BMP transducers have been identified, including KRAS, NOX and NRF2. With these transducers, BMP is able to serve as a co-modulator in reactive oxygen species (ROS), suggesting an indirectly influence on cancer progression through microsatellite instability status and chemoresistance [16–19]. Owing the modulation ability in cancer development, there are several research focusing on regulation mechanism of BMP pathway. Among these research, long non-coding RNA working through ceRNA mechanism on microRNA occupies a very special role [20–22].

Among various lncRNAs, FGD5-AS1 caught our attention for its influence on tumorigenesis and tumor development. According to sequences published on NONCODE (http://www.noncode.org/), we predicted putative proteins encoded by lncFGD5-AS1 using ORF Finder (https://www.ncbi.nlm.nih.gov/orffinder/) followed by analyzing the codon substitution frequency scores (CFS). The non-coding nature of lnc FGD5-AS1 was confirmed by no ORF was larger than 200 nt and CFS was negative [23, 24]. Although role of FGD5-AS1 in oral cancer, periodontitis, glioma, and hepatocellular cancer has been fully investigated [25–28],. the function of FGD5-AS1 in gastric cancer still lacks enough evidence.

In this study, we firstly confirmed that lncFGD5-AS1 inhibited metastasis, proliferation and EMT in vitro and in vivo by regulating miR-196a-5p/SMAD6 to inhibit BMP pathway, which suggested the potential of FGD5-AS1 as a candidate treatment target.

## Methods

### Cell culture and cell transfection

MKN74, MKN45 and HEK-293 cell lines were purchased from ATCC (American Type Culture Collection, Manassas, USA) though Genechem (Shanghai, China) with the cat.no. GCD01854, GDC01855 and GCD0156566. And all cells were grown in Roswell Park Memorial InstituteRPMI-1640 medium with 10% fetal calf serum at 37 °C and in a 5% CO2 incubator. All cells were confirmed without any mycoplasma contamination tested by the Myco-Lumi™ luminescence mycoplasma detection kit (C0298S, BiYunTian, China) annually. (The most recent detection was carried out on Aug 2020.)

Lentivirus particles were designed and purchased from Genechem (Shanghai, China), including LV-FGD5-AS1, LV-MiR-196a-5p-precursor, LV-anti-MiR-196a-5p, and controls. The vectors were as follows: Ubi-MCS-SV40-EGFP-IRES-puromycin used for FGD5-AS1 overexpressed, hU6-MCSUbiquitin-EGFP-IRES-puromycin was used for MiR-196a-5p up-regulation, hU6-MCSCMV- EGFP was used for MiR-196a-5p down-regulation. Lentivirus transfection was performed according to the manufacturer’s instructions. Lentivirus was transfected with Enhance Liquid and Polyberene as ratio provided by Genechem.

Mimics of miR-196a-5p was designed and purchased from GenePharma (Shanghai, China). Mimics was transfected with lipo2000 (Invitrogen, ThermoFisher, USA).

The BMP pathway inhibitor, LDN-193189 from Axon Medchem (Netherlands), was dissolved with DMSO in 100 nM and added into cells culturing in 6-well plates for 10 h before cell lysed for protein collection.

### Clinical and tissue samples

The clinical tissue cDNA chip was purchased in Shanghai Outdo Biotech Co., Ltd. (CGt No: cDNA-HStmA060CS01; Lot No:96*R100-M-201703xx-xx,), containing 30 pairs of cancer and para-cancer normal tissue. Para-cancer normal tissue was defined as the tissue more than 2 cm away but less than 3 cm away from the tumor edge. The 96-well plate was centrifuged before being performed with usage of SYBR Green real time PCR MasterMix (Takara (Japan)) and FGD5-AS1 primer. All the patients were gastric carcinoma diagnosed with pathological analysis. The tumor node metastasis (TNM) stage were assessed according AJCC Cancer Staging Manual [29].

### Cell migration assay

Cell metastasis was examined using transwell assay, co-culture chambers were purchased from BD Biosciences (San Jose, CA, USA). Serum-free medium are placed in top chambers, and medium containing 10% FBS was added to the bottom chambers. Cells were evenly suspended in top chambers. Then the 6-well plate with chambers were cultured for 24 h at 37 °C with 5% CO2. After incubation, the non-metastasis cells were gently removed from the top wells with a cotton-tipped swab and the chambers were fixed with methanol for 30 min. The chambers were then stained with crystal violet for another 30 min. Cell counting was facilitated by photographing the membrane through amicroscope (Zeiss) under a × 10 objective lens.

### Cell proliferation assay

Cell counting Kit-8 (Genview, GK3607-100 T) was used to detect cell proliferation. 5000 suspended cells were planted in 96-well plate with complete medium containing 10%FBS. After the suspended cells adherence in 8 h, 10% CCK solution was added in the well. Incubating for another 40 min, then microplate reader was used to test the OD value at 450 nm, which will indicate the proliferation of cells.

### Quantitative reverse transcription-PCR

Trizol purchased from Invitrogen (Calsbad, USA) was used to extract total cell RNA as standard protocol. RNA concentration was tested by Nanodrop (Invitrogen, USA). PrimeScriptTM RT Master Mix kits were used to synthesis cDNA; and Taqman MirNA assay kit (designed for microRNA) and SYBR® Select Master Mix kit (designed for total RNA) were used for RT-qPCR analysis on Bio-Rad CFX96 qPCR instrument (Bio-Rad,Hercules, CA). All these kits were purchased from Takara (Japan). β-actin and U6 were used as reference genes.

### Western blot assay

RIPA lysis (ThermoFisher, USA) was used for extracting total cell protein. Protein concentration was tested by BCA assay (ThermoFisher, USA). Equal amounts of protein were separated by SDS-PAGE gel as regular protocol. And the protein was transferred from gel to a PVDF membrane (Millipore). Then the PVDF membrane was blocked in 5% BSA-TBST (Sigma, USA) for 2 h at room temperature, followed by primary antibody at 4-degree overnight. The membrane was washed 10 min 3 times with TBST, followed by secondary antibody at room temperature for 1 h. After another washing cycle, the membrane was visualized by ultra-sensitive ECL kit. Results was calculated by Target protein/ β-actin based on band intensity tested by ImageJ.

The related antibodies used were as follows:

E-cadherin (CST, Boston, USA,14472), N-cadherin (CST, Boston, USA, 13116), Vimentin (CST, Boston, USA, 5741), Snail (CST, Boston, USA), MMP9(CST, Boston, USA), smad2/3((abcam, USA, ab202445), p-smad2/3 (CST, Boston, USA),BMP4 (abcam, USA, ab124715),P-SMAD1/5/8(CST, Boston, USA, 13820), Anti-SMAD1/5/8 antibody (abcam, USA,ab80255), and TGF-BETA1 (abcam, USA, ab179695), SMAD6(SANTA CRUZ, USA, sc-25,321).

### Dual luciferase reporter assay

Luciferase reporter assay. The binding sites of 3’UTR in SMAD6 were analyzed by Targetscan and were amplified by polymerase chain reaction (PCR) and inserted into the vector, which was designed and purchased by Genechem (Shanghai, China). Then transfected the 3’UTR plasmids and miR-196-5p mimics in to HEK 293 cells. Besides that, Renilla luciferase expression plasmid was co-transfected (Genechem Shanghai, China) as transfection control in all groups. After 24 h, cells were lysed in 250 μl of Passive Lysis Buffer (Promega) and 20 μl were used to measure luciferase activity with the Luciferase Assay System (Promega). The different groups as follow: PGL3-NC + microup, PGL3-NC + microNC, PGL3-*SMAD6*MUT + micro up, PGL3-*SMAD6*MUT + micro NC, PGL3-*SMAD6*WT + micro up, PGL3-*SMAD6*WT + microNC. Each group has 6 parallel holes, and the assay was repeated for 3 times.

### Fluorescence in situ hybridization (FISH)

RNA-FISH was used to test location of lncRNA FGD5-AS1. The florescence probes of FGD5-AS1 were purchased from Genechem (Shanghai, China). 18S rRNA was the probe for cytoplasmic control. For analysis the MKN-74 cells were cultured on slides. The slides were fixed in absolute ethyl alcohol for 15 min (Sigma), and then cells were treated by cold 0.1% Triton-100x for 15 min before hybridized with probes overnight at 37 °C. After washing with SSC/0.3%Tween20 buffer, the coverslip was dyed with DAPI and fluorescence test was conducted with laser scanning confocal microscope (Leica Application Suite, Germany). DAPI channel was set at 410-485 nm showing the location of nucleic; Cy3 was chosen as cytoplasmic dye with excitation wavelength at 548-681 nm. The merged image was established with Leica Application. Scale bar was chosen as 50 μm showing in the Fig. 2a.

### Tumor xenografts in mice

Twelve 4-weeks old female J;NU Homozygous for Foxn1nu mice were obtained from the Medical Laboratory Animal Center of Xi’an Jiao Tong University and averagely separated into four cages randomly. The initial body weights of mice were 20 ± 0.4 g (mean ± STD). All nude mice were housed under specific pathogen-free conditions and the order of the cages changed every week by staff member to avoid position influence.

To test the function of lncFGD5-AS1 in vivo, we randomly separated these mice into lncFGD5-AS1 overexpressed group and control group (3 mice/cage, 2 cages/group). The stable transfected Lv-FGD5-AS1 MKN45 and LV-control MKN45 cells were counted and suspended in 50% matrigel (USA, Corning) followed by injecting subcutaneously with 106cells 0.1 ml in each mouse. These mice were observed under standard SPF housing condition and the length, width and height of the tumor were tested every 3 days following randomly order for 5 weeks.

Before final test, two mice from control group were sacrificed for cachexia (severe body weight loss with body weight of 12.3 g & 15.8 g) at the early of 4th week; two mice from FGD5-AS1 overexpressed group were excluded from the experiment for the tumor didn’t grow successfully. At final test time, each group had four 10-weeks old mice. Mice were anesthetized with isoflurane while harvesting tumor tissue and sacrificed with cervical dislocation. The xenograft tumor tissues were harvested and weighted immediately followed by extracting protein and RNA from these tissues according to the common protocol. The protein and RNA were employed for further western blot and RT-QPCR test.

The experiment was in accordance with the regulation of the Ethics Committee of Xi’an Jiao Tong University and the Guide for the Care and Use of Laboratory Animals published by the US National Institutes of Health. The animal test was approved by the Ethics Committee of Xi’an Jiao Tong University and the ethics document was numbered as NO.G-271.

### Statistics methods

The data in this article are shown as the means ± SEM. Data was collected from at least three independent experiments. Paired Student-test was used to test the difference between paired groups. Unpaired Student-test was used to test the difference between un-paired groups. Chi-square test was used to test the difference for binary variables. Wilcoxon paired test was used to test differences between paired patients’ samples because the difference value doesn’t obey normal distribution. Graphpad (GraphPad Software, La Jolla, CA, USA) was used to do the statistics analysis. Image J was used to collect intensity from western blot Fig. *P* < 0.05 was marked as *; *P* < 0.01 was marked as **; *P* < 0.005 was marked as***, *P* < 0.001 was marked as ****.

### Bioinformatics analysis

The target gene and binding site of miR-196a-5p was predicted by TargetScan (http://www.targetscan.org/vert_72/). The competition lncRNA and binding site of miR-196a-5p was predicted by Starbase (http://starbase.sysu.edu.cn/starbase2/index.php).

The Kaplan–Meier analysis was performed using the online Kaplan–Meier Plotter (http://www.kmplot.com) to estimate relapse-free survival curves of the 875 gastric cancer patients, and the median threshold was used as the cut-off point for the high and low groups of FGD5-AS1 expression.

## Results

### Lower level of lncRNA FGD5-AS1 indicated a worse prognosis

**Fig. 1:**
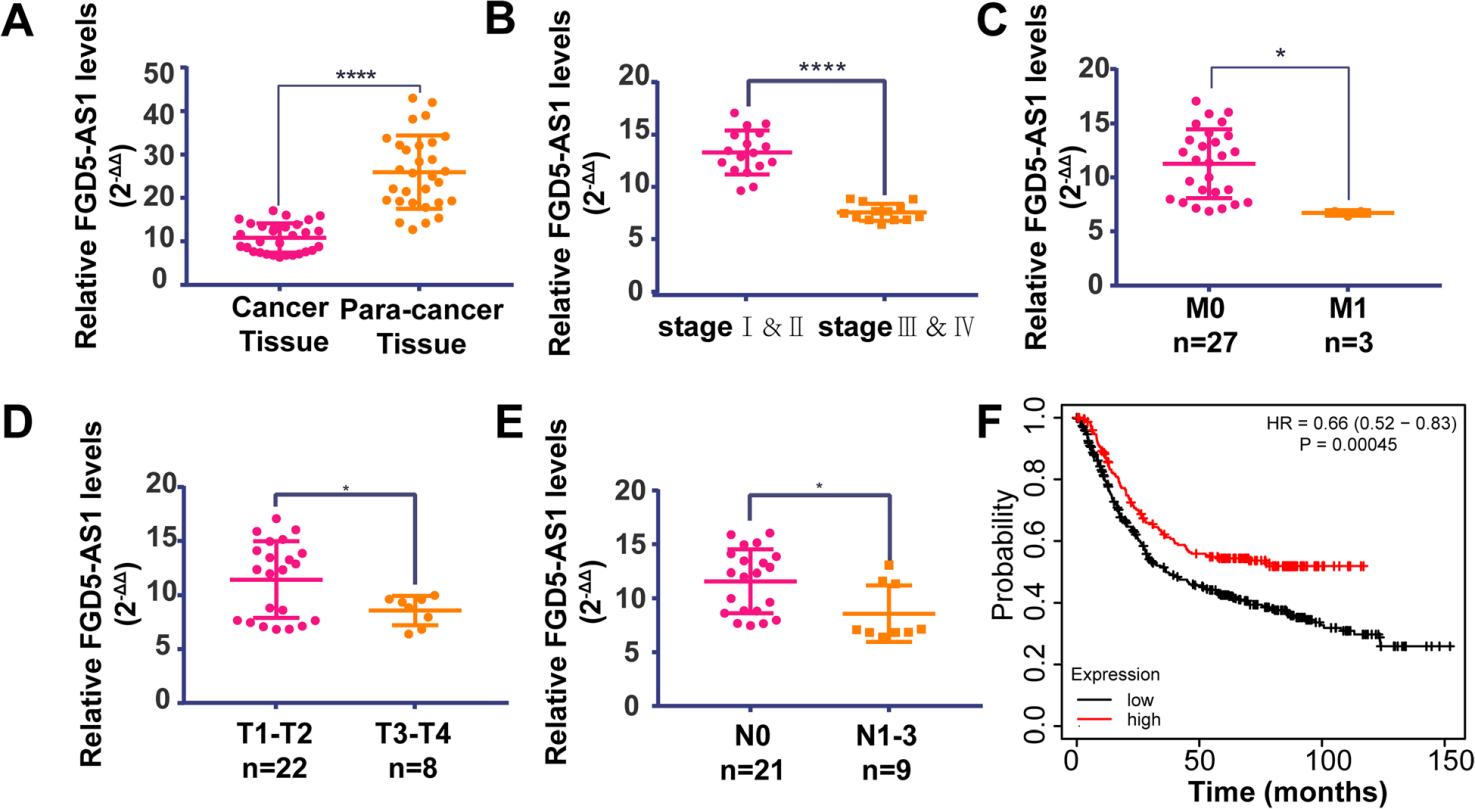
Prognose analyze of FGD5-AS1 in patients’ tissue. **a** QRT-PCR for FGD5-AS1 in 30 gastric cancer tissue cDNA chips comparing expression level in para-cancer tissue and cancer tissue. (Wilcoxon paired test was used to test the difference between paired samples because the difference value doesn’t obey normal distribution. * = significant, * *P* < 0.05; **, *P* < 0.01,***P < 0.005; **** < 0.001, Student t test). **b** &**c**&**d**&**e** According to the mean value of FGD5-AS1(presented by 2^-ΔΔ, Mean value: 10.81); patients were divided into two groups: high FGD5-AS1 expression and low FGD5- AS1 expression group. The expression level of FGD5-AS1 shows remarkably relation with anatomic stage/pronostic groups stages. Meanwhile comparing T1–2 vs T3–4, N0 vs N1–3, M0 vs M1 we can clearly summarize that the high level of FGD5-AS1 correlates with serious TMN stage. (* = significant,* *P* < 0.05; **, *P* < 0.01***, *p* < 0.005;****, *p* < 0.001; Student t test); **f**) Kaplan-Meier survival analysis of 875 gastric cancer patients downloaded from the TCGA database for the expression of FGD5-AS1 (Log Rank; *p* = 0.00045). The median expression of FGD5-AS1 was regarded as a cut off point. The red line represented the high expression group and the blank line represented the low expression group

To investigate the potential relationship between FGD5-AS1 and the cancer process, we compared FGD5-AS1 levels in 30 patients’ tumor tissue and para-cancer normal tissue. From Fig. 1a and Table 1, we can clearly summarize that cancer tissue had significantly lower FGD5-AS1 expression than normal tissue.

**Table 1:** Expression of FGD5-AS1 in cancer and para-cancer tissue

	Low expression	High expression	*P*-value
Para-cancer tissue	6	24	0.0149
Cancer tissue	15	15	

^a^ The median expression level was used to distinguish high or low expression

We separated all patients into high expression and low expression two groups by FGD5-AS1 expression levels in cancer tissue. The median expression level was regarded as the cut-off point. From Fig. 1 b&c&d&e Fig. S1, we can summarize that lower expression of FGD5-AS1 had strong relation with higher anatomic stage/prognostic tissue and higher TNM stage. FGD5-AS1 influence moderately within the M stage, poorly within tumors that were ≥ 5 cm and differentiated degree. (Table 2 & Fig. 1 b&c & d & e) Besides that, an overall survival analysis based on FGD5-AS1 expression level was carried on in 875 gastric cancer patients (Data downloaded from TCGA database). As shown in Fig. 1f, higher FGD5-AS1 expression level had consistently higher survival probability from 0 months to 150 months (Log Rank *p* = 0.00045; HR = 0.66), which strongly indicated the positive relationship between FGD5-AS1 expression and a better prognosis.

**Table 2:** Correlation of FGD5-AS1 expression with clinical and pathological characteristics of gastric cancer patients

Variables	Number of patients (30)	Low expression (15)	High expression (15)	***P***-value
**Sex**				
Male	24	12	12	0.99
Female	6	3	3	
**Age**				
≤ 60	18	8	10	0.4561
> 60	12	7	5	
**Tumor diameter**				
< 5 cm	12	8	4	0.1360
> 5 m	18	7	11	
**T**				
1–2	8	1	7	**0.0132**
3–4	22	14	8	
**N**				
0	9	3	6	**0.0053**
1–3	21	12	9	
**M**				
0	27	12	15	0.0679
1	3	3	0	
**Serous membrane infiltration**				
No	11	2	9	**0.008**
Yes	19	13	6	
**Differentiation degree**				
Moderately differentiated	12	7	5	0.4561
Poorly differentiated	18	8	10	
**Anatomic stage/prognostic groups**				
I&II	13	3	10	**0.0099**
III&IV	17	12	5	

^a^ The median expression level was used to distinguish high or low expression

^b^ *p* value was calculated by chip-square test

In general, a lower expression of FGD5-AS1 strongly indicated worse prognosis and shorter survival time.

### LncRNA FGD5-AS1 mainly locates in the cell cytoplasm and combined with miR-196a-5p

**Fig. 2:**
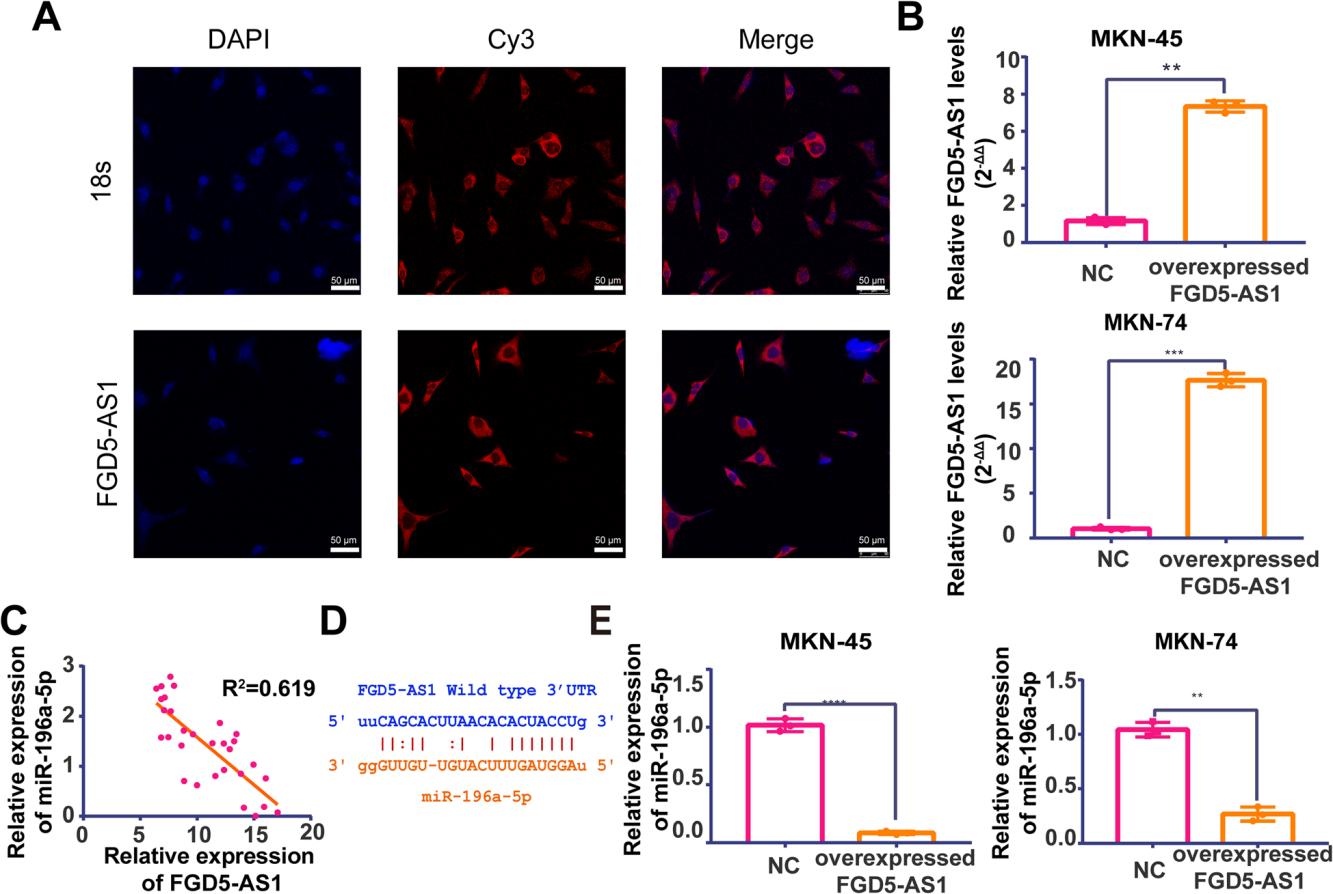
FGD5-AS1 locates in cytoplasm and binds with miR-196a-5p. **a** Intracellular localization of FGD5-AS1 in MKN-45 was carried out through RNA-FISH assay. The location of 18 s was used as positive control; Nuclei was stained by DAPI; 18 s rRNA (cytoplasmic positive) and FGD5-AS1 were labeled with CY3.(Scla Bar = 50 μm); **b **The overexpression of FGD5-AS1 was tested in MKN45 and MKN74, respectively (Data was expressed as mean ± SD (*n* = 3) * = significant, * *P* < 0.05; **, *P* < 0.01,***P < 0.005; **** < 0.001, Student t test); **c** & **d** The level of miR-196a-5p and the level of FGD5-AS1 in patients’ tissues has significantly negtive trend. (y = − 0.1905x + 3.448; R^2^ = 0.6197; Y: miR-196a-5p expression level expressed by 2^-ΔΔ^; X: FGD5-AS1 expression level expressed by 2^-ΔΔ^). **e** The level of miR-196a-5p was tested by QRT-PCR in overexpressed FGD5-AS1 cell lines; U6 was used as control. (Data was expressed as mean ± SD (*n* = 3) * = significant, * *P* < 0.05; **, *P* < 0.01,***P < 0.005; **** < 0.001, Student t test);

For positioning FGD5-AS1, we perform RNA-FISH in FGD5-AS1 highest expression cell line MKN-45. From Fig. 2a, we can see FGD5-AS1 labeled by red fluorescence mainly located in cytoplasm rather than nucleus, which indicates lncRNA FGD5-AS1 high likely acts through ceRNA theory. To support this point, we downloaded analyze of FGD5-AS1 location in various cell lines from LNCATLAS atabase (https://lncatlas.crg.eu/). Results also show FGD5-AS1 had a significantly higher expression in cytoplasm. (Fig. S2) According to bioinformatics analysis results, miR-195a-5p high likely was the target gene of FGD5-AS1.To verify FGD5-AS1 and miR-196a5p expression level correlation, we tested miR-196a-5p in the same 30 patients’ tumor tissue. As shown in Fig. 2c, there is an apparent negative trend between FGD5-AS1 and miR-196a-5p expression levels. To further test expression correlation between miR-196a-5p and FGD5-AS1, we overexpressed FGD5-AS1 in MKN-45 and MKN-74 followed by testing miR-196a-5p mRNA level in these cell lines. (Fig. 2b&e) The negative expression correlation strongly supported the ceRNA relationship between miR-196a-5p and FGD5-AS1.

### Overexpressed FGD5-AS1 sharply inhibits cancer cells proliferation, migration and epithelial-mesenchymal transition process

**Fig. 3:**
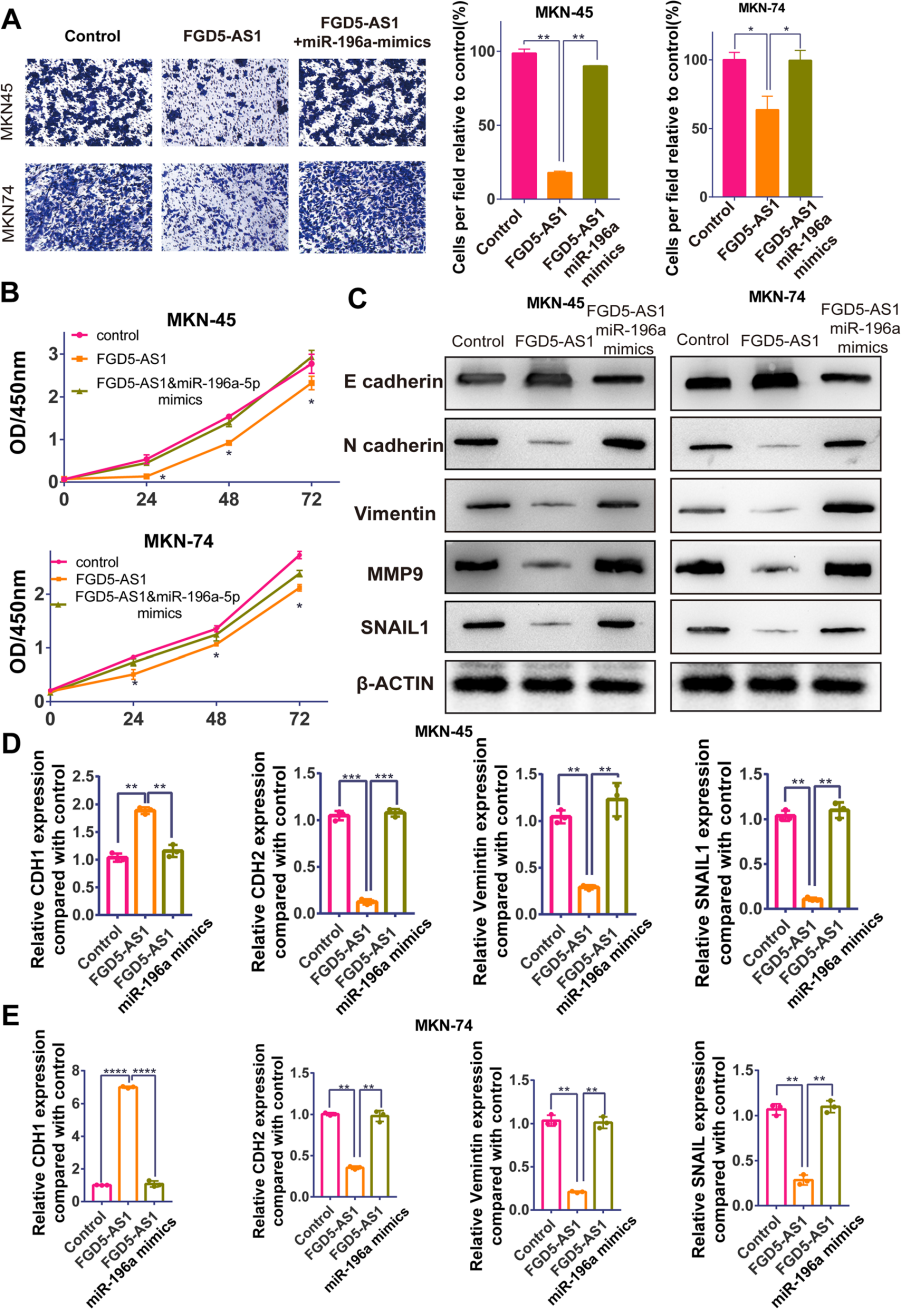
Overexpressed FGD5-AS1 sharply inhibits cancer cells proliferation, migration and epithelial-mesenchymal transition process. **a** Transwell assay was utilized to visually confirm metastasis inhibition function of overexpressing FGD5-AS1, and co-transfected with miR-196a-5p mimcs was used as rescue test to confirm the inhibition acting through ceRNA with miR-196a-5p. The statistics of per field cells were also illustrated the same result; Cells were calculated under microscope;The average number unber 9 fields was regarded as migaration cell amounts. (Data was expressed as mean ± SD (*n* = 3) * = significant, * *P* < 0.05; **, *P* < 0.01,***P < 0.005; **** < 0.001, Student t test); **b**) CCK-8 assay was used to test the proliferation change after FGD5-AS1 overexpressed. FGD5-AS1 group had the lowest cell viability; while co-overexpressed FGD5-AS1 and miR-196a-5p had no statistic difference compared with control group; Data was expressed as mean ± SD (*n* = 6) * = significant, * *P* < 0.05; **, *P* < 0.01,***P < 0.005; **** < 0.001, Student t test). **c** The protein level of EMT markers, E-cadherin, N-cadherin, Vimentin, MMP9 and SNAIL1, were tested by western blot in FGD5-AS1 overexpressed MKN-45 & MKN74 cell lines (The first and second lane counted from left). Co-overexpressing miR-196a-5p group used to test role of miR-196a-5p. The third lane shows no difference with control group. The original blots were shown in Fig. S4. **d** & **e** The RNA level of EMT makers, E-cadherin, N-cadherin, Vimentin, SNAIL1, were tested by western blot in FGD5-AS1 overexpressed MKN-45 & MKN74 cell lines (Data was expressed as mean ± SD (*n* = 6) * = significant, * *P* < 0.05; **, *P* < 0.01,***P < 0.005; **** < 0.001, Student t test)

Subsequently, we focused on FGD5-AS1 function in cancer metastasis and proliferation. We employed a transwell assay and a CCK-8 assay to test its effects on cancer metastasis and proliferation, respectively. As illustrated by Fig. 3a&b, migrated cells and OD/450 nm had sharply reduced in both MKN-45 and MKN-74 after overexpressing FGD5-AS1. In comparison, overexpressed FGD5-AS1 + miR-196a-5p mimics exhibited no differences with the control group. These results indicated that function of FGD5-AS1 can be rescued by increasing miR-196a-5p level, which strongly supported and indicated the ceRNA relationship and downstream role of miR-196a-5p. Based on the close relationship between epithelial–mesenchymal transition (EMT) and cancer initiation, we employed western blot and RT-qPCR to test epithelial and mesenchymal markers. As shown in Fig. 3c-f, overexpressed FGD5-AS1 inhibited expression of mesenchymal markers (N-cadherin and Vimentin) and promoted expression of epithelial markers (E-cadherin) with decrease of transcription factor SNAIL1 and migration marker MMP9.

In conclusion, reduction of mesenchymal markers and increase of epithelial makers indicated that a higher FGD5-AS1 induced tumor cells transiting from mesenchymal status into epithelial status, which will lead to cancer cell metastasis and proliferation.

### miR-196a-5p suppressed SMAD6/BMP pathway to influence EMT process

**Fig. 4:**
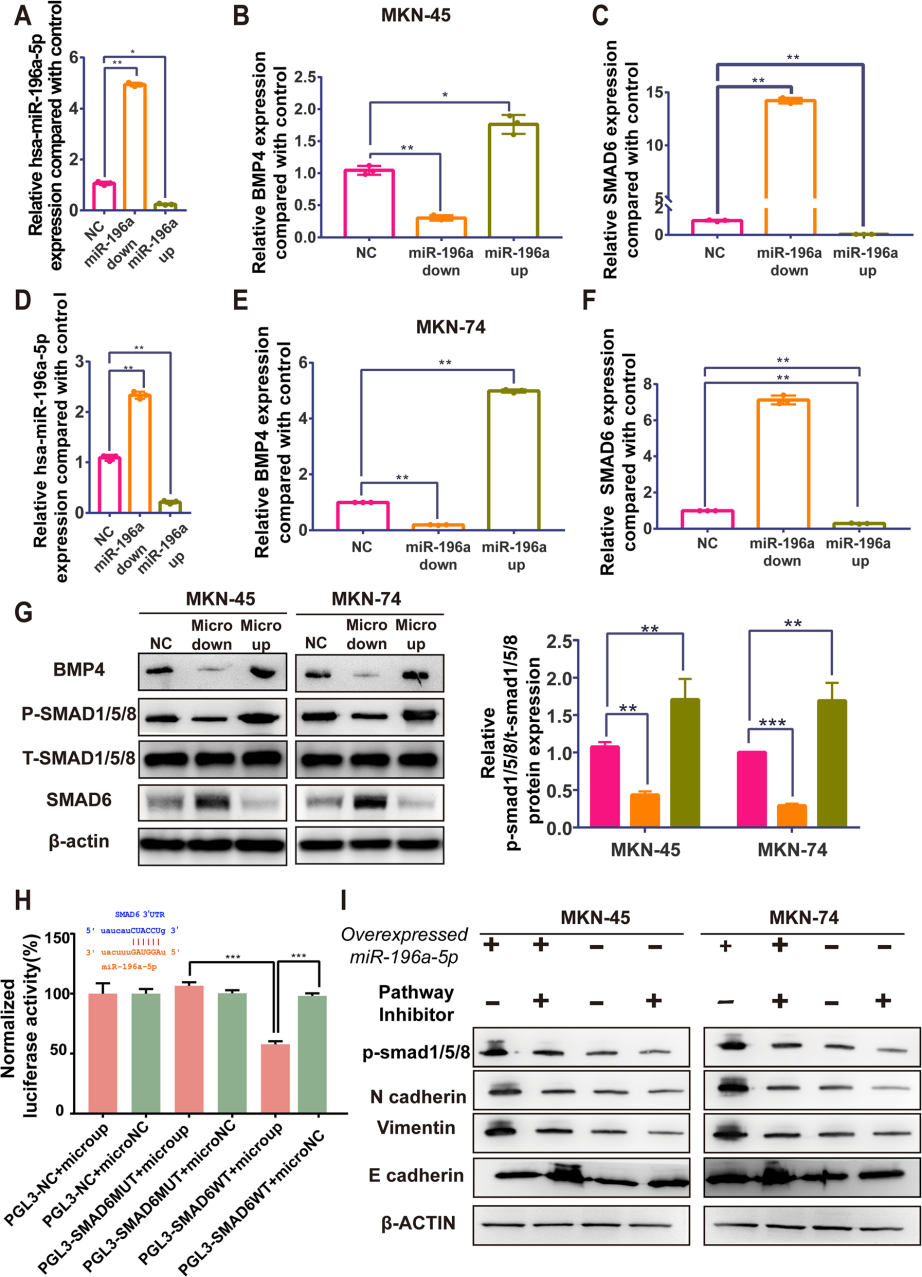
miR-196a-5p endocompetes with SMAD6 acting on BMP pathway to influence EMT process. **a** &**d** The overexpression and downregulation of miR-196a-5p was tested in MKN45 and MKN74, respectively (Data was expressed as mean ± SD (*n* = 3) * = significant, * *P* < 0.05; **, *P* < 0.01,***P < 0.005; **** < 0.001, Student t test); **b**) &**c**) &**e**) &**f**) After overexpressed and down regulated miR-196a-5p, the mRNA level of SMAD6 changes negtively with miR-196a-5p; while the mRNA level of BMP4 changes positively with miR-196a-5p. (Data was expressed as mean ± SD (*n* = 3) * = significant, * *P* < 0.05; **, *P* < 0.01,***P < 0.005; **** < 0.001, Student t test); **g** Western blot for down and up regulated miR-196a-5p in MKN45 and MKN74 to test protein level of BMP pathway markers, BMP-4, t-smad1/5/8, p-smad1/5/8 and smad6. The figure shown that the level of BMP4 and p-smad1/5/8 changes positively with the level of miR-196a-5p. The ratio of p-smad1/5/8 and t-smad1/5/8 indicated that miR-196a-5p influenced activation of BMP pathway. The original blots were shown in Fig. S5A &S5B. **h** Dual-luciferase report assay. Wild-type and mutated 3’UTR-binding site was cloned in luciferase-reported plasmid and mimcs of miR-196a-5p or nc of miR-196a-5p was transferred with these plasmid into HEK293 cells. Compared with other groups, PGL3-SMAD6WT+ micro-up group normalize luciferase activity decrease by about 40%. The significant reduce prove that the level of SMAD6 decrease when co-transfected with miR-196a-5p. (Data was expressed as mean ± SD (*n* = 6) * = significant, * *P* < 0.05; **, *P* < 0.01,***P < 0.005; **** < 0.001, Student t test). **i** Effect of BMP pathway inhibitor LDN-193189. Western blot for EMT markers, E-cadherin, N-cadherin, Vimentin, and BMP pathway factors in MKN-45 and MKN-74 respectively as the following groups: micro-up, microup+LDN, microNC+DMSO and micro NC+ LDN. The original blots were shown in Fig. S6.

To the exact mechanism of miR-196a-5p, we overexpressed and downregulated miR-196a-5p by lentivirus in MKN-74 and MKN-45, respectively. The overexpression and downregulation efficiency were shown in Fig. 4 a&d.

Based on the bioinformatic analysis, SMAD6 is the most likely target gene of miR-196a-5p and shared the same combination sites with lncFGD5-AS1. (Fig. 2d & Fig. 4h) To verify the suppression of miR-196a-5p on BMP pathway, expression of BMP4, t-smad1/5/8, p-smad1/5/8/ and smad6 were tested in miR-196a-5p overexpressed and downregulated cell lines by western blot and RT-PCR respectively. (Fig. 4 b, c, e, f, g) Results shown that SMAD6 exhibited a reverse trend with regulation of miR-196a-5p. Meanwhile, we employed dual-luciferase assay to confirm the combination between miR-196a-5p and between 3’UTR of *Smad6.* Predicted binding sites was cloned in luciferase-reported plasmid. After co-transfecting with miR-196a-5p mimics in smad6 wild-type sequences, the level of luciferase activity sharply reduced to 60.75% compared with the mutated binding sites. (Fig. 4h) Similar reduction of luciferase activity also shown in dual-luciferase report assay carried in GES-1. (Fig. S3).

Besides that, the ratio of phosphorylated smad1/5/8 (p-smad1/5/8) and total smad1/5/8 (t-smad1/5/8) confirmed that the phosphorylation of BMP pathway influenced by miR-196a-5p. To further confirm the necessary role of the BMP pathway in the EMT inhibition process, we employed the whole BMP pathway inhibitor LDN193189 to rescue the inhibition. By comparing the first and third lane of Fig. 4i, overexpressing miR-196a-5p sharply increased N-cadherin & Vimentin level and decrease the E-cadherin level. In comparison, the overexpression of miR-196a-5p makes no influence on EMT markers in the blocking group. Surprisingly, the combination of BMP pathway block and miR-196a-5p overexpression resulted in a general increase of E-cadherin, which may be reasoned by other pathways influencing by microRNA and pathway block.

In general, miR-196a-5p combines with 3’UTR of SMAD6 to promote BMP pathway. A high level of FGD5-AS1 overwhelmingly combined with miR-196a-5p inducing overexpression of smad6 which inhibited the BMP pathway and further inhibited the EMT process.

### LncFGD5-AS1 suppressed tumor proliferation and EMT in vivo

**Fig. 5:**
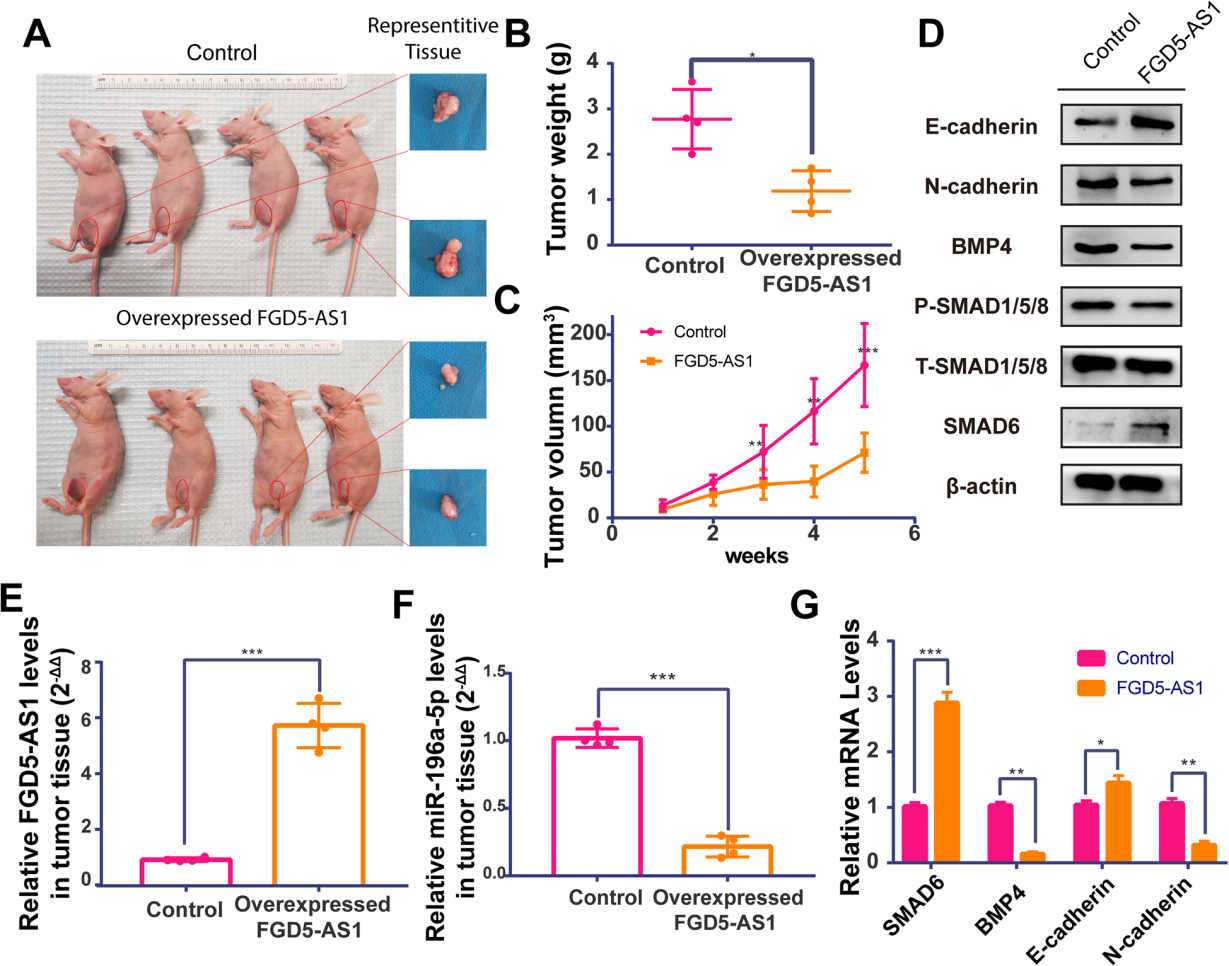
Overexpression of lncRNA FGD5-AS1 inhibits BMP pathway and EMT process in gastric cancer xenograft mouse model. **a** The macroscopic observation of subcutaneous tumor lesions and representative isolated tumor tissues from nude mice bearing xenograft tumor with normal MKN-45 cells (control group) and Overexpressed-FGD5-AS1 MKN45 cells injected. (Scale bar is shown in the image. **b** The average tumor weights in nude mice. (* = significant,* *P* < 0.05; **, *P* < 0.01,***P < 0.001; Student t test). **c** The growth curve of tumor volume. (* = significant,* *P* < 0.05; **, *P* < 0.01,***P < 0.001; Student t test). **d e**&**g** Western blot and PCR was used to show the expression of E-cadherin, N-cadherin, BMP4, t-smad1/5/8, p-smad1/5/8 and SMAD6 in tissues derived from xenograft tumors. (*N* = 4,* = significant,* *P* < 0.05; **, *P* < 0.01,***P < 0.001; Student t test) The original blots were shown in Fig. S7. **e**&**f** RT-PCR was used to test the expression of FGD5-AS1 and miR-196a-5p in tissues derived from xenograft tumors. (*N* = 4,* = significant,* *P* < 0.05; **, *P* < 0.01,***P < 0.001; Student t test)

To examine the effect of LncRNA FGD5-AS1 on gastric cancer in vivo, we developed a nude mouse xenograft tumor model by subcutaneous injecting FGD5-AS1 overexpressing MKN45 and NC-MKN45. (Fig. 5a) Qrt-pCR showed that the tumor tissue in overexpression group had higher FG5-AS1 level and lower miR-196a-5p expression. (Fig. 5e & f) After observing these tumors for 5 weeks, we found that the growth speed of tumor size in control group is higher than FGD5-AS1 group. (Fig. 5b) Meanwhile, the weight and final volume of harvested tumor tissue from mice in control group is heavier than FGD5-AS1 group. (Fig. 5c) In the 5th week, the tumor volume and tumor weight of the control group were 166.74 + 39.28 mm^3^ (*n* = 4) and 2.78 + 0.57 g (*n* = 4), respectively. In contrast, the tumor volume and tumor weight of the FGD5-AS1 overexpressed group were 71.05 + 18.63 mm^3^ (*n* = 4) and 1.18 + 0.39 g (*n* = 4), respectively. Data were shown as mean ± std.

To verify the function of the lncFGD5-AS1/miR-196a-5p/SMAD6/BMP axis in vivo, we employed western blot and QRT-PCR in harvested tumor tissue. As shown in Fig. 5 d&g, the mRNA and protein levels of E-cadherin and SMAD6 were markedly increased with a decrease of N-cadherin and BMP4 in xenograft tumors from the FGD5-AS1 group. Meanwhile, the ratio of p-smad1/5/8 and t-smad1/5/8 exhibits markedly decrease in FGD5-AS1 group, which indicated the inhibition of BMP pathway.

## Discussion

**Fig. 6:**
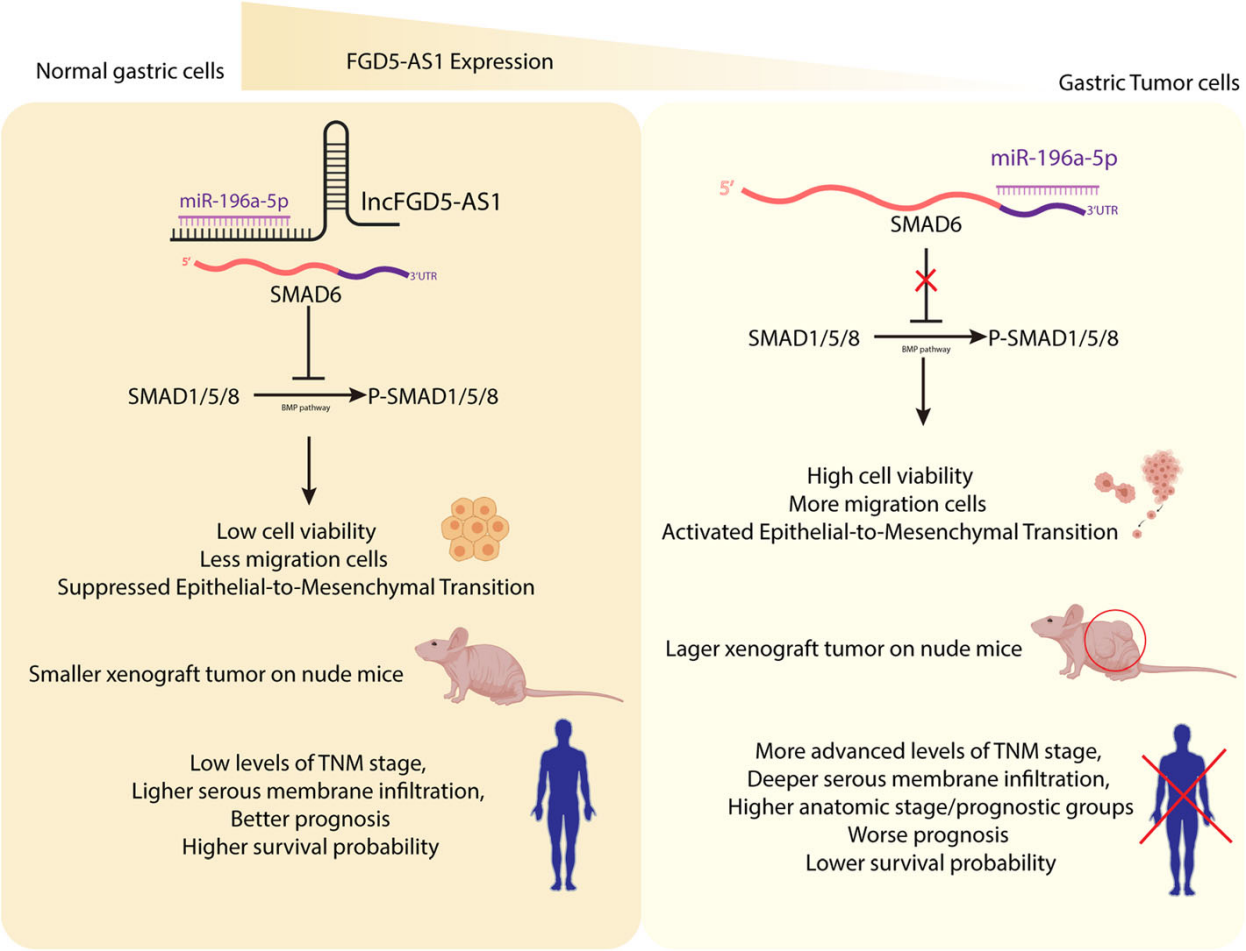
Graphic Abstract. This research verified that a lower level of lncFGD5-AS1 could induce higher tumor cell viability and more migration tumor cells in vitro and larger tumor volume and tumor growth rate in vivo, which led to more advanced levels of TNM stage, deeper serous membrane infiltration, higher anatomic stage/prognostic groups, worse prognosis, and lower survival probability in gastric cancer patients. In mechanism, this article also investigated that lncFGD5-AS1 worked through regulating miR-196a-5p/SMAD6/BMP axis on the EMT process and served as a potential therapeutic candidate for gastric cancer. This figure was created with BioRender.com

LncRNAs have been proved as an essential regulator and indicator in cancer. In this article, we found a high lncRNA FGD5-AS1 level in gastric cancer suppressing epithelial to mesenchymal transition and indicating a good prognosis. We clarified this conclusion through the analysis of patient samples, proliferation & metastasis characteristics in cell lines, and tumor xenograft nude mice. Firstly, we found that more advanced levels of TNM stage, deeper serous membrane infiltration, higher anatomic stage/prognostic groups, and worse prognosis strongly related to lower expression of FGD5-AS1. Besides that, the overexpressed FGD5-AS1 group had less migration cells, lower cell viability, lower mesenchymal markers expression, and higher epithelia markers expression. To localize FGD5-AS1, we performed RNA-FISH in MKN-45 and further confirm it with the lncRNA location database. Unfortunately, limited by the access to proper patients’ samples, we haven’t performed RNA-FISH locating FGD5-AS1 in biopsies samples. The cytoplasm location indicates FGD5-AS1 is likely working through a ceRNA mechanism. Subsequently, we employed a dual-luciferase report assay confirming the ceRNA relationship between FGD5-AS1 and miR-196a-5p. Mimics of miR-196a-5p can rescue influence of FGD5-AS1 in vitro. In pathway level, FGD5-AS1 was confirmed working through rescuing the suppression of miR-196a-5p on SMAD6, BMP pathway inhibitor. As a regulator of oxidative factors, BMP4 is upregulated under oxidative stress which will facilitate cancer progression. The upregulated BMP4 is also able to reciprocally stimulate ROS pathway and act on cancer progression [14, 17, 30, 31]. Furthermore, tumor xenograft nude mice model proved that FGD5-AS1 could suppress tumor growth and EMT process through BMP pathway. The full schematic was shown in Fig. 6.

Prognostic markers could select patients who need more intensive treatment. Lacking proper prognostic markers is a common concern in gastric cancer treatment. Currently, clinical doctors prefer using symptom including tumour size, grade, and lymphovascular invasion to assess the patients’ survival. However, gastric cancer is a highly heterogeneous disease indicating patients suffering similar symptom will have extremely different prognosis and survival status.

indicating that traditional evaluation system has remarkable limits in predicting patients’ outcome [3–7]. To replace the conventional symptom prognosis evaluation system, extensive efforts have been dedicated to investigating non-coding RNA markers.

Our results strongly proved that higher FGD5-AS1 is a strong independent prognostic predictor of better survival. Interestingly, results provided by Li et al. [32] in their recent article showing that higher lncRNA FGD5-AS1 also performed a negative role in gastric cancer treatment through promoting chemoresistance. Similar differences also exist in difference tissue type, other research carried in colorectal cancer [33], lung cancer [34], and oral cancer [25]. The huge difference between these two kinds of conclusions illustrating the diversification in lncRNA function and heterogeneity in lncRNA expression [11, 35]. Mostly acting through the ceRNA mechanism, lncRNA directly working on the complementary microRNA followed by indirectly influencing the transcriptional expression level of downstream coding genes [36–38]. As an element in the dynamic network of non-coding RNA mechanism, lncRNA FGD5-AS1 owes bilateral function in tumor progression which requires more detailed research. Meanwhile, lncRNA have not been employed in clinical practice even though numerous previous researchers have focused on therapeutic potential of non-coding RNA [5, 6, 39, 40]. Their instability and tiny amount limited the accurate detection. Besides that, non-coding RNA has such wide influence in body which means it will have high sensitivity but extremely low specificity. The clear relationship between detectable abnormal value and typical clinical disease is still lack in clinical practice. Unfortunately, we have not solved these problems in this article. Nevertheless, we truly illustrated mechanism of lncRNA FGD5-AS1’s function in gastric cancer. And we believe this will be basis for further clinical utilization of non-coding RNA.

## Conclusion

In conclusion, as shown in the full schematic (Fig. 6) this research verified that a lower level of lncFGD5-AS1 could induce higher tumor cell viability and more migration tumor cells in vitro and larger tumor volume and tumor growth rate in vivo, which led to more advanced levels of TNM stage, deeper serous membrane infiltration, higher anatomic stage/prognostic groups, worse prognosis, and lower survival probability in gastric cancer patients.

In mechanism, this article also investigated that lncFGD5-AS1 worked through regulating miR-196a-5p/SMAD6/BMP axis on the EMT process and served as a potential therapeutic candidate for gastric cancer.

## Supplementary Information

**Additional file 1.**

**Additional file 2.**

**Additional file 3.**

**Additional file 4.**

**Additional file 5.**

**Additional file 6.**

**Additional file 7.**

## Data Availability

All data generated or analyzed during this study are included in this published article except for gastric cancer survival analysis data downloaded from the TCGA database. The datasets are available in the http://www.kmplot.com. LncRNA expression data was downloaded from https://www.ncbi.nlm.nih.gov/geo/ (GSE29272, GSE163988 and GSE158662).

## Abbreviations

BMP: Bone morphogenetic proteins
BMPR: Bone morphogenetic proteins receptors
CeRNA: Competing endogenous RNA
CFS: Codon substitution frequency scores
EMT: Epithelial-to-mesenchymal transition
p-smad1/5/8: Phosphorylated small mothers against decapentaplegics 1/5/8 complex
ROS: Reactive oxygen species
SPF: Specific pathogen-free
T-smad1/5/8: Total small mothers against decapentaplegics 1/5/8 complex
