# Supplementary figures and images for: A high level of lncFGD5-AS1 inhibits epithelial-to-Mesenchymal transition by regulating the miR-196a-5p/SMAD6/BMP axis in gastric Cancer
Source: BMC Cancer. 2021 Apr 23;21:453. doi: 10.1186/s12885-021-08192-x (PMC8066889; doi:10.1186/s12885-021-08192-x)

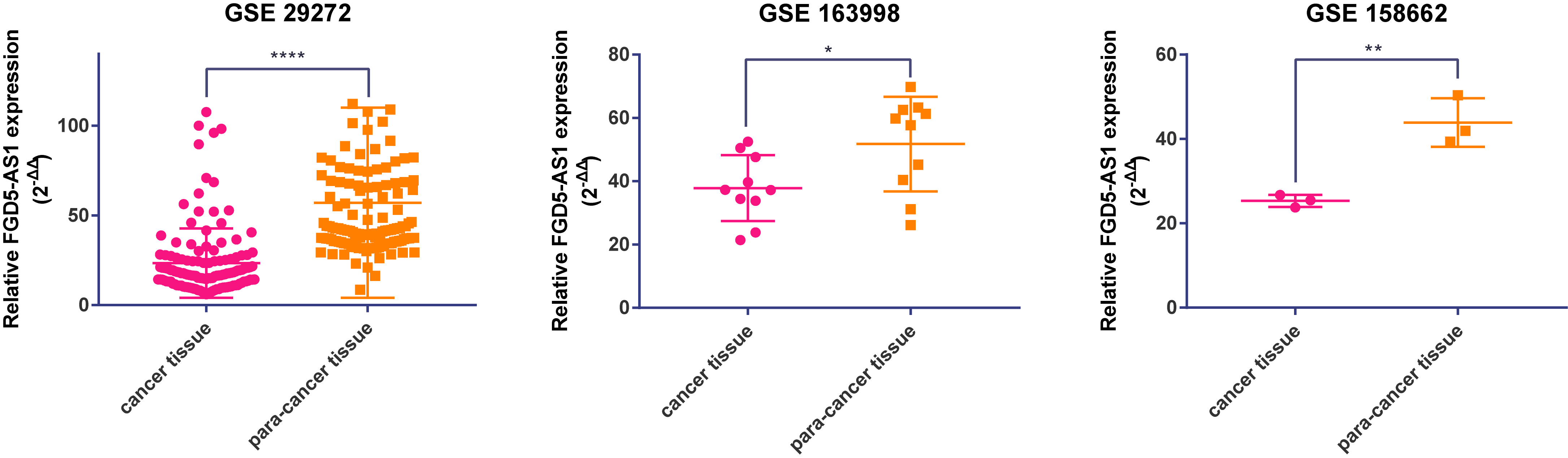

Supplement: Supplementary file 1 — Additional file 1. [file 12885_2021_8192_MOESM1_ESM.tif]

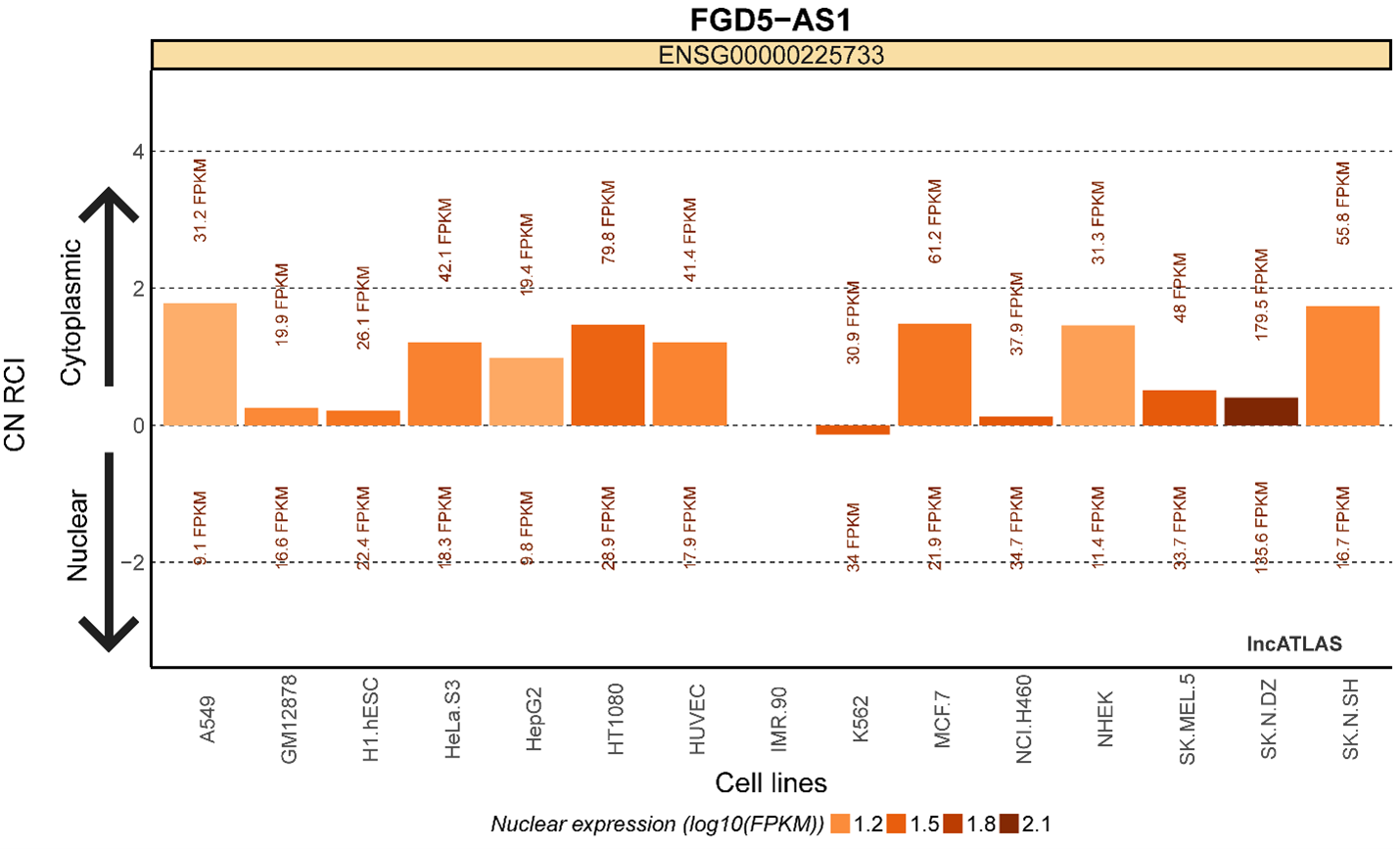

Supplement: Supplementary file 2 — Additional file 2. [file 12885_2021_8192_MOESM2_ESM.tif]

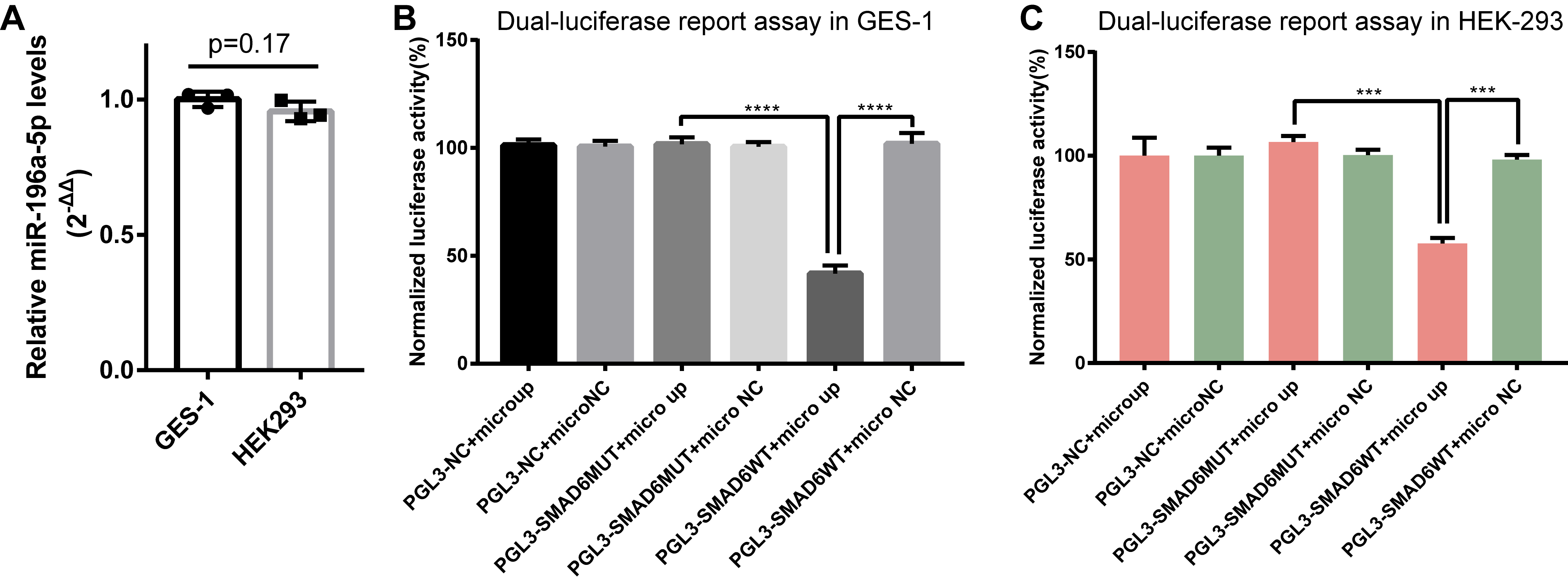

Supplement: Supplementary file 3 — Additional file 3. [file 12885_2021_8192_MOESM3_ESM.tif]

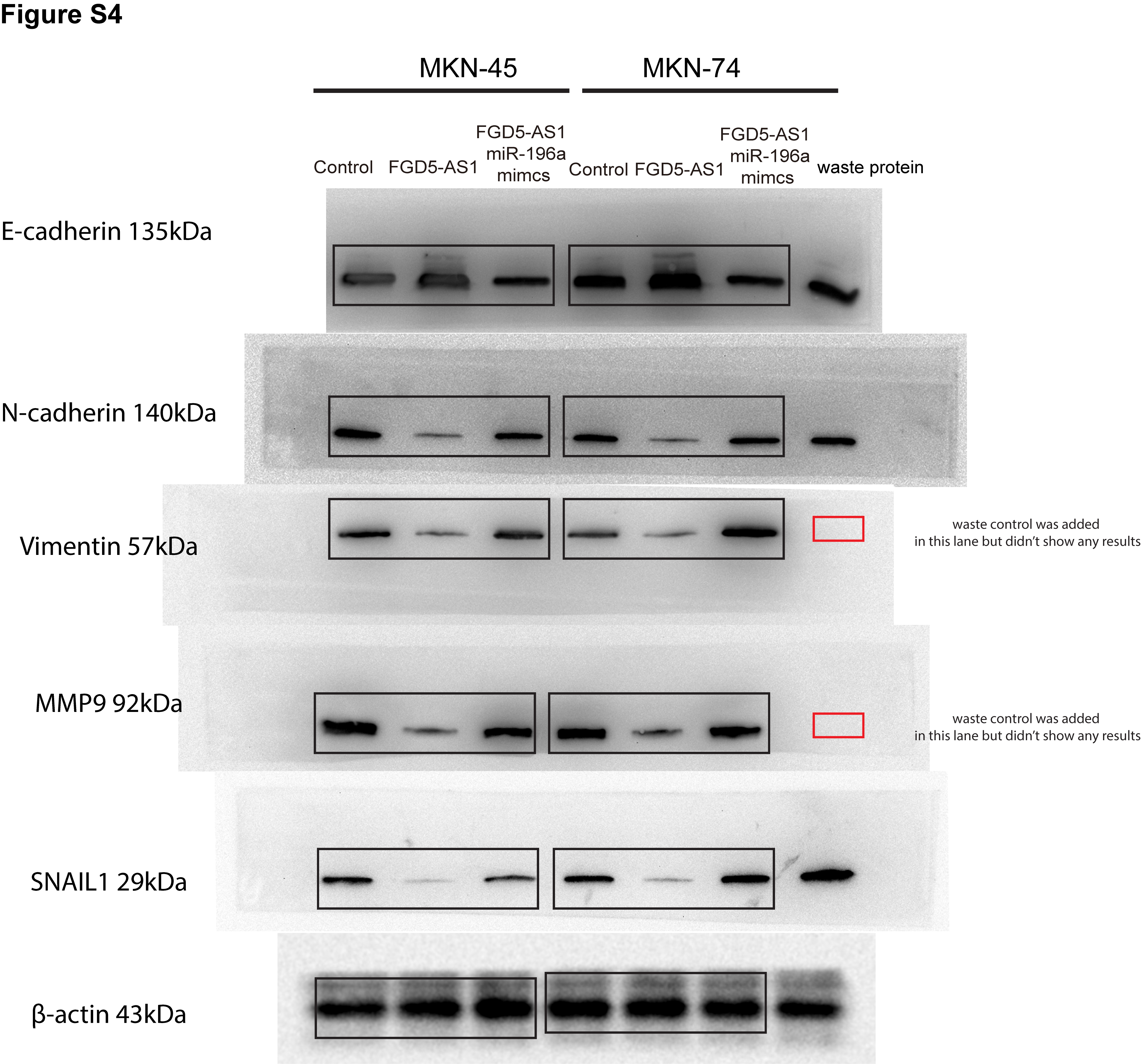

Supplement: Supplementary file 4 — Additional file 4. [file 12885_2021_8192_MOESM4_ESM.tif]

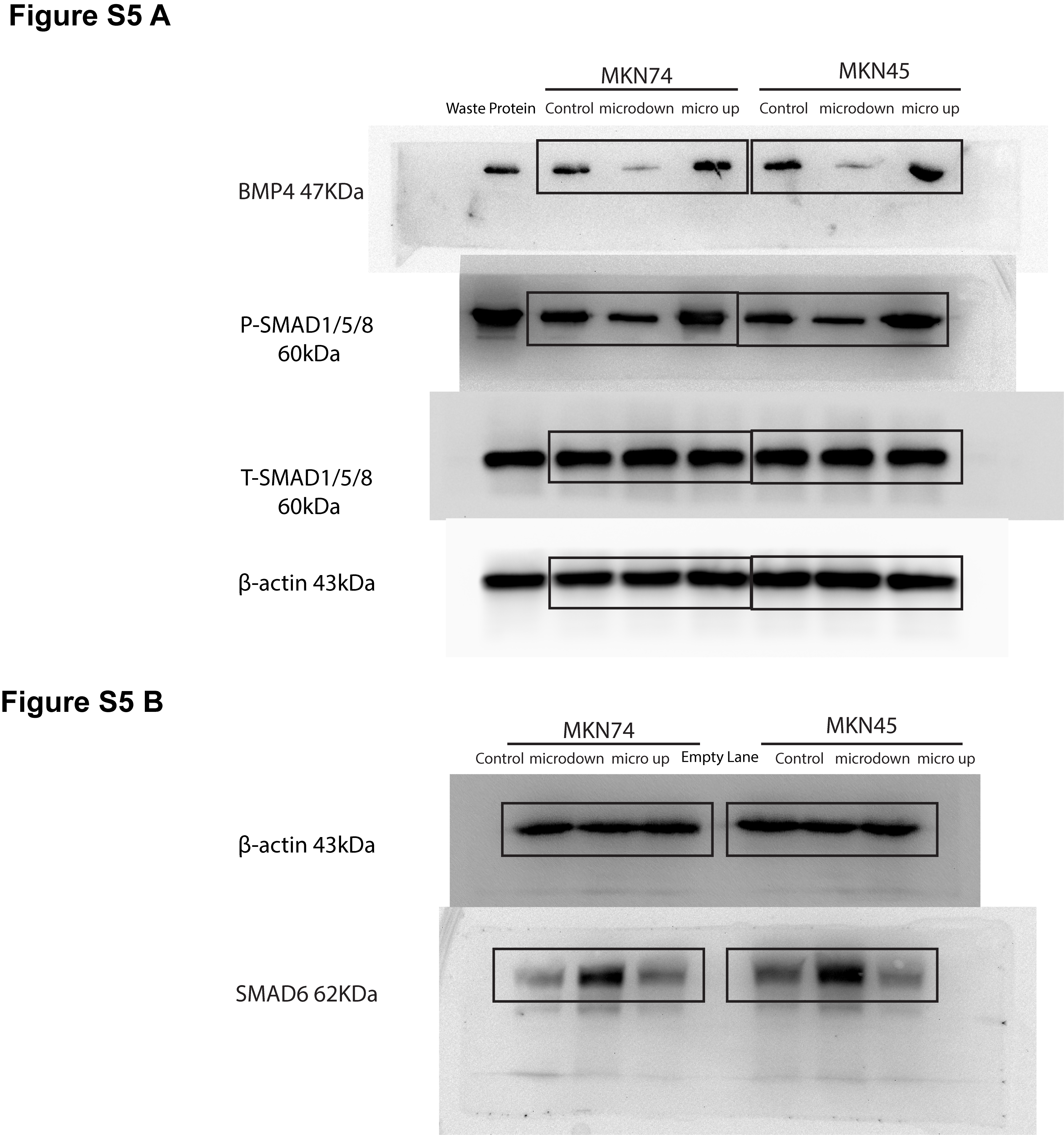

Supplement: Supplementary file 5 — Additional file 5. [file 12885_2021_8192_MOESM5_ESM.tif]

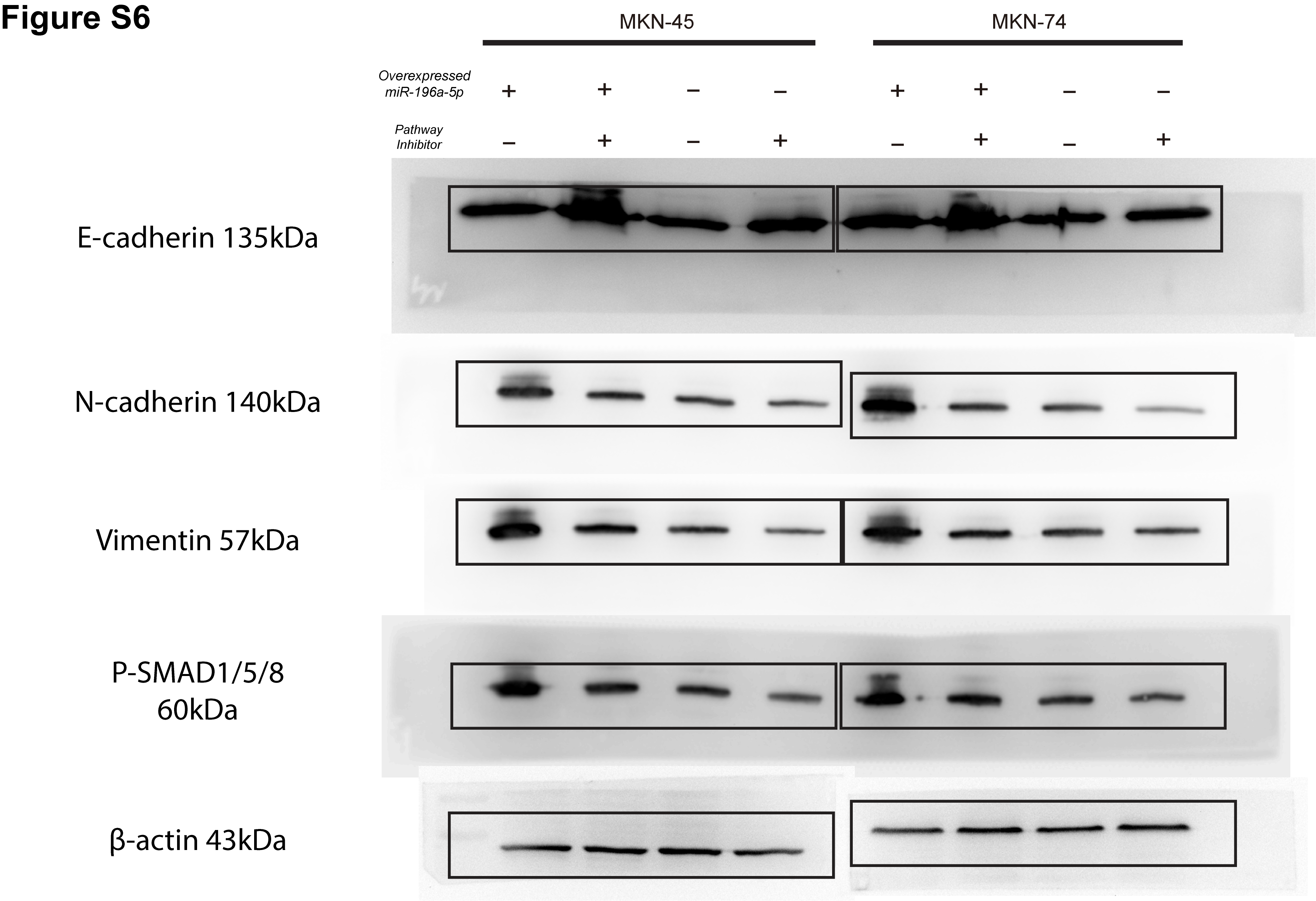

Supplement: Supplementary file 6 — Additional file 6. [file 12885_2021_8192_MOESM6_ESM.tif]

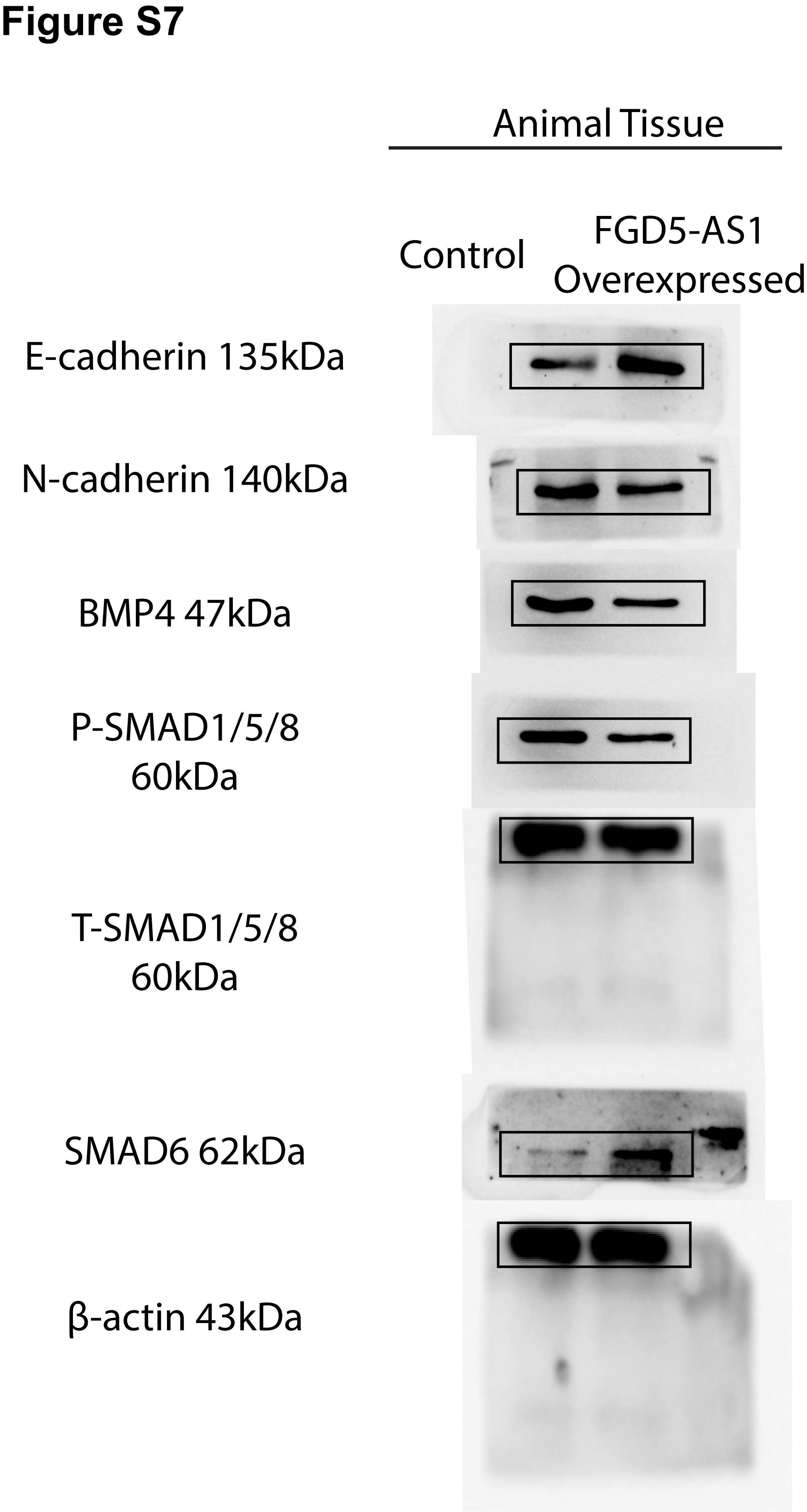

Supplement: Supplementary file 7 — Additional file 7. [file 12885_2021_8192_MOESM7_ESM.tif]
